# Colorectal Carcinoma Affected Patients Are Significantly Poor Responders Against the Oncogenic JC Polyomavirus

**DOI:** 10.3389/fimmu.2021.632129

**Published:** 2021-05-25

**Authors:** Elena Torreggiani, Ilaria Bononi, Silvia Pietrobon, Elisa Mazzoni, Giovanni Guerra, Carlo Feo, Fernanda Martini, Mauro Tognon

**Affiliations:** ^1^ Department of Medical Sciences, School of Medicine, University of Ferrara, Ferrara, Italy; ^2^ Department of Translational Medicine, School of Medicine, University of Ferrara, Ferrara, Italy; ^3^ Clinical Laboratory Analysis, University-Hospital of Ferrara, Ferrara, Italy

**Keywords:** colorectal carcinoma, polyomavirus, JCPyV, oncogenic, antigen, antibody, prevalence, mimotope

## Abstract

**Background:**

Many investigations reported the association between human tumors and JCPyV, a polyomavirus with oncogenic potential. The association has been supported by studies that found JCPyV footprints in CRC and gliomas of different types. Indeed, JCPyV footprints including its nucleic acids and Tag oncoprotein have been revealed in CRC tissues.

**Methods:**

Herein, sera from colorectal carcinoma (CRC) affected patients and healthy individuals (HS), employed as control, were analysed for immunoglobulin G (IgG) antibodies against specific JCPyV viral capsid protein 1 (VP1) antigens. The investigation was carried out employing an innovative immunological assay. Indeed, an indirect enzyme-linked immunosorbent assay (ELISA) with JCPyV VP1 mimotopes was used. JCPyV VP1 mimotopes consisted of synthetic peptides mimicking VP1 epitopes.

**Results:**

Sera from CRC affected patients, evaluated using indirect ELISAs with synthetic mimotopes, showed a significant lower prevalence of IgG antibodies against JCPyV VP1 mimotopes (26%) compared to HS (51%), p<0.005. These data were confirmed by another method, the hemagglutination inhibition (HAI) assay. Altogether these results, i.e. the prevalence of serum IgG antibodies against JCPyV VP1 mimotopes from patients with CRC is approximately 50% lower than in HS, are of interest.

**Discussion:**

Our data suggest that patients with CRC are significantly poor responders against JCPyV VP1 antigens. It is possible that CRC patients are affected by a specific immunological deregulation. This immunological dysfunction, revelled in CRC patients, may account for their predisposition to the colorectal carcinoma onset.

## Introduction

Colorectal carcinoma (CRC) is a tumour arising at high frequency in distinct human populations. CRC can be a fatal tumour (1, 2), and is responsible of approximately 10% of all malignancies in humans (3). So far, several risk factors associated with CRC aetiology have been revealed, including infectious agents, biology background and lifestyle (4–7).

Among viruses with oncogenic potential, different polyomaviruses have been investigated for their association with human tumors. JCPyV is a neurotropic and oncogenic polyomavirus, which has a pivot role in the onset of multifocal leukoencephalopathy (PML). In addition, human tumors of different hystotypes tested JCPyV-positive. Indeed, many investigations found JCPyV associated with cancers of the central nervous system (CNS), such as gliomas of different types and colorectal carcinomas (8–10). However, other studies reported negative data (11).

JCPyV is considered an opportunistic pathogen (12) which infection occurs in the first years of life. JCPyV is present in the adult population with a prevalence ranges from 50 to 60% (13) and the prevalence reaches 70% in the elderly (14). JCPyV is constituted by a circular double strand DNA of 5.13 Kb (15). Its genome is characterized by two main coding regions named early and late regions. The viral oncoproteins large T (Tag), small t (tag) antigens, together with the multifunctional agnoprotein (agno) are encoded by the early region sequences, whereas the three viral capsid proteins VP1, VP2 and VP3, which are structural proteins, are encoded by the late region. Moreover, JCPyV genome has a non-coding region (NCCR), with a regulatory function (16).

JCPyV has been investigated for its role in the development of gastrointestinal cancers, including CRC (17, 18). Many independent studies reported the detection of JCPyV nucleic acids or proteins, and in particular the oncoprotein Tag, in various human tissues including adenomatous polyp tissues and colorectal adenocarcinomas; they have also been found in normal tissues and adjacent non-cancerous tissues (19).

JCPyV, with its viral oncoprotein Tag, is able to induce chromosomal instability in colonic cells. This mechanism of action favors gross chromosomal rearrangements, loss of heterozygosity and aneuploidy which may facilitate, during the multistep phases of the tumorigenesis, the cell transformation of colorectal cells (20). In addition, other investigations detected JCPyV DNA, Tag and the JCPyV-specific microRNA 5p (miR-J1-5p) in CRC biopsies, being their presence associated with the tumor development (7, 21–23). However, other studies did not identify JCPyV DNA sequences in hyperplastic polyps/adenoma and adenocarcinoma and normal tissues (11, 24–26). The reasons for these contrasting data reported by different investigations are not known. However, these contrasting results may be due to different protocols employed during the sample collection and processing, JCPyV testing, different genetic background of patient populations (27).

The association between JCPyV and CRC has been poorly studied (28–31). To date, investigations in this field show inconclusive and conflicting, results about the putative involvement of JCPyV in CRC. Discrepancies of data on the association between JCPyV and CRC could be due to the cross-reactivity of the JCPyV proteins, used as antigens in immunological tests. Indeed, JCPyV antigens share an extensive amino acid homology with BK (BKPyV) and simian virus 40 (SV40), which belong to polyomavirus family, too (13, 32–35). In this context, it should be recalled that CRC, with an incidence and mortality rate expected to increase more than 60% up to 2030, represents a global emergency (3). Conflicting results, emerging from different studies, suggest that the association between JCPyV and CRC deserves further investigations.

Recently, an innovative immunological test to reveal serum JCPyV-antibodies has been reported. This test is based on the use of two synthetic peptides, named mimotopes used as antigens of JCPyV viral capsid protein 1 (VP1) in an indirect enzyme-linked immunosorbent assay (ELISA) (13, 36).

The main goal of this investigation is to verify JCPyV antibodies in sera from CRC affected patients employing two immunological tests, i.e. an innovative indirect ELISA with JCPyV mimotopes and the Haemagglutination Inhibition (HAI) assay. The combination of the two tests was employed to confirm the specificity of the results (13, 37).

## Materials and Methods

### Serum Samples

Blood samples were obtained from the Clinical Laboratory Analysis, University Hospital of Ferrara. Specifically, samples (n=53) were from patients affected by colorectal carcinoma (CRC) at the time of collection and healthy subjects (n=89; HS). Briefly, whole blood was collected anonymously, coded with indications of age, gender and pathology. After clot formation, blood samples were centrifuged at 800 g for 10 min., whereas the resulting supernatant serum was carefully removed. Approximately 500 µl ÷ 1 ml of serum was collected from each blood sample. Sera were then divided into aliquots and stored at -80°C until the time of the analysis.

### Ethical Statement

The County Ethical Committee of the University Hospital of Ferrara approved this study, number 151078. All CRC patients and HS subjects gave written informed consent to the scientific research at the time of the hospital admission, in compliance with the Declaration of Helsinki.

### JCPyV VP1 Mimotopes

As previously described (13), computer-assisted analyses enabled to select two specific peptides from the JCPyV viral capsid protein 1 (VP1) sequences. Given the high homology among viruses belonging to the polyomavirus family, the sequence of selected JCPyV specific peptides was compared with the same region of the highly homologous BKPyV and SV40 polyomaviruses and with the same region of other less homologous viruses (http://blast.ncbi.nlm.nih.gov).

JCPyV specific peptides named VP1 K and VP1 N have the following amino acid sequences:

1. VP1 K: NH2–KSISISDTFESDSPNRD—COOH (17 a.a, 60–76);
2. VP1 N: NH2–LMNVHSNGQATHDNGAGK—COOH (18 a.a, 118–135).

Previous indirect ELISA results (13, 36), indicate that JCPyV VP1 K and VP1 N peptides did not cross-react with the BKPyV and SV40 hyperimmune sera used. An unrelated polyomavirus human peptide, the human neuropeptide S (hNPS), was employed as a negative control antigen. The a.a. sequence of hNPS has been previously reported (38, 39). Peptides, synthesized by standard procedures, were purchased from UFPeptides s.r.l. (Ferrara, Italy).

### Indirect Enzyme-Linked Immunosorbent Assay

JCPyV synthetic peptides, named VP1 K and VP1 N were developed as antigens in an indirect ELISA which was standardized to identify specific antibodies against JCPyV in human sera (13, 36, 40).

#### Peptide Coating

Ninety-six-well flat bottom plate (Nunc-Immuno plate PolySorp; Thermo Fisher Scientific, Milan, Italy), was coated with 5 μg of the selected peptide for each well diluted in 100 μl of Coating Buffer (Candor Bioscience, Weissensberg, Germany). The plate was incubated at 4°C for 16 h allowing the peptide to cover the bottom well completely.

#### Peptide Blocking

The plate was rinsed three times with Washing Buffer (Candor Bioscience) using a microplate washer (model Wellwash 4MK2; Thermo Electron Corporation, Vantaa, Finland). This procedure removes uncoated peptides. Blocking was performed with 200 μl/well of the Blocking Solution (Candor Bioscience) at 37°C for 90 min.

#### Serum Sample Addition

After the blocking step the three washes with Washing Buffer (Candor Bioscience) were repeated. Then, 100 μl of human serum samples under investigation were added to the plate. Moreover, 100 μl of the following sera were added in other wells of the same plate and used as controls: (i) an immune rabbit serum containing anti-JCPyV antibodies represented the positive-control; (ii) human immune sera anti-SV40 antibodies, (iii) human immune sera anti-BKyV antibodies, and (iv) three human serum samples which were found to be JCPyV negative in previous studies (35), represented the negative controls. Each human sample was diluted 1:20 in Low Cross Buffer (Candor Bioscience) and analysed three times. The plate was incubated at 37°C for 90 min.

#### Secondary Antibody Addition

After incubation, a new triple rinsing cycle was repeated as described above. Then, the secondary antibody solution was added to each well. The solution consists of a goat anti-human or anti-rabbit Ig-G heavy and light chain specific peroxidase‐conjugate (Calbiochem-Merck, Darmstadt, Germany) diluted 1:10,000 in Low Cross Buffer (Candor Bioscience) was incubated at room temperature for 90 min.

#### Dye Treatment and Spectrophotometric Reading

Following incubation, the plate was rinsed three times with the Washing Buffer (Candor Bioscience) and then treated with 100 μl of 2,2′- azino‐bis 3-ethylbenzthiazoline-6-sulfonic acid solution (Sigma-Aldrich, Milan, Italy), which reacts with the peroxidase enzyme, enables the color reaction. After 45 min at room temperature, 100 μl of citric acid 0.1 M was used to block the colorimetric reaction. The spectrophotometer (model Multiskan EX; Thermo Electron Corporation) was employed to read the plate at a wavelength (λ) of 405 nm. Color intensity in wells was determined by optical density (OD) reading that corresponds to the amount of immune complexes formed, which in turn depends on the quantity of specific antibody against JCPyV is present in samples tested.

#### Cut-off Determination

The cut-off value in each assay was determined using an OD reading of the three negative control sera added three times to the standard deviation (+3 SD) (13, 35, 36). The three JCPyV negative control sera were chosen from those below the cut-off value calculated using second–degree polynomial regression by plotting the ranked net OD individual values for VP1 K and VP1 N peptides, as previously published for MCPyV and BKPyV virus-like particles (VLPs) (41, 42). Sera with JCPyV VP1 antibodies were considered positive upon reacting to both peptides from the late region.

Result reproducibility was determined by performing three independent replicates of the experiment carried out by different operators. The specificity and sensitivity of the results observed with the indirect ELISA, were confirmed by comparing the immunological data with the results obtained by the haemagglutination inhibition (HAI) assay.

### JCPyV Haemagglutination (HA) and Haemagglutination-Inhibition (HAI) Assays

JCPyV titre was assessed by haemagglutination (HA) with a solution of human erythrocytes, group 0, Rh+. These erythrocytes agglutinate in the presence of a specific concentration of JCPyV virions, forming a network that keeps red cells in suspension. On the contrary, in the absence or with a low virion concentration, red cells precipitate and form a red spot on the bottom of the well (37, 43). Serial dilutions of the viral working stock purchased from the American Type Culture Collection (ATCC, VR-1583), were carried out in 96 well round bottom plates (Thermo Fisher Scientific, Milan, Italy). Specifically, the titration of JCPyV was based on serial 1:2 dilutions of the virus in Dulbecco’s Phosphate Buffered Saline, DPBS 1X (Lonza, Milan, Italy) from 1:10 to 1:5120, in 100 μL of final volume. Then, 50 μL of 1% erythrocytes, were added to each viral dilution. Plates were incubated at 4°C, whereas the HA titre was read 4 h later when the control, represented by erythrocytes in DPBS only, had completely sedimented on the well bottom. The HA titre was determined based on the highest viral dilution that causes a complete haemagglutination (13, 37). The viral titre established by HA assay was 3.2 x 10^3^ haemagglutinating units (HU), corresponding to 3.2 x 10^7^ plaque-forming unit in 1 ml (PFU/ml).

Haemagglutination-inhibition (HAI) assay tests the capacity of JCPyV immune serum samples from CRC and HS to inhibit the haemagglutination ability of the virus. Serial dilutions (1:16, 1:32, 1:64, 1:128) of serum were employed to calculate the antibody titre. Sera were heated at 56°C for 30 min and treated with 0.1 M sodium periodate (NaIO_4_) to remove non-specific inhibitors. Specifically, 30 μL of serum diluted 1:2 was added to 15 μL of NaIO_4_, in 96 well round bottom plates (Thermo Fisher Scientific, Milan, Italy) and incubated at room temperature for 30 min. Then, 15 μL of 5% glycerine was added to each serum. Serum serial dilutions in DPBS 1x, from 1:16 to 1:128, were mixed with 8 haemagglutinating units (H.U.) of JCPyV as antigen. Mixtures were maintained at room temperature for 1 h. Then, 1% of human erythrocytes solution, group 0, Rh+, was added, then plates were incubated at 4°C for 4 h. As previously described (37, 44), the HAI titre was calculated based on the highest dilution of each immune serum sample that completely abolish the viral HA.

### Statistical Analysis

The differences between the prevalence of anti-JCPyV antibodies in the sera of the different groups of subjects studied (CRC and HS) were analyzed statistically. χ2 test with Yates’ correction was employed to compare binary variables. Fisher’s exact test was used to compare the prevalence of anti-JCPyV antibodies between CRC patients and HS subjects. The serologic profile (OD) of serum antibody reactivity to JCPyV mimotopes was statistically analyzed using a one-way t-student test.

The comparability of age and gender distribution between CRC and HS was evaluated by student’s t-test. P < 0.05 values were considered statistically significant. All statistical analyses were carried out using GraphPad Prism 6 software (GraphPad software, La Jolla, CA, USA).

## Results

### Identification of JCPyV Antibodies in Patients Affected by Colorectal Carcinoma Through an Indirect E.L.I.S.A.

Sera were from colorectal carcinoma affected patients (CRC, n = 53) healthy subjects (HS, n = 89), employed as the control group. The two cohorts had similar median age distribution and gender (**Table 1**), were evaluated for IgG antibodies (abs) reacting to JCPyV VP1 mimotopes, known as VP1 K and VP1 N. To this aim, an indirect ELISA was developed using the aforementioned synthetic peptides as viral antigens (13, 36), whereas a human peptide, hNPS, which is not associated to polyomavirus proteins, was the control (38, 44). CRC and HS serum samples were diluted 1:20, then evaluated for their reactivity to JCPyV VP1 epitopes in indirect ELISAs.

**Table 1:** Prevalence of serum immunoglobulin G antibodies reacting to JCPyV viral capsid protein 1 (VP1) mimotopes in indirect ELISA.

Human serum	N. of samples	Median age ± SD	Male (%)	N. of positive samples (%)		
				VP1 K	VP1 N	VP1 (K+N)
CRC	53	69 ± 12	33 (62)	15 (28)	16 (30)	14 (26)**
HS	89	61 ± 11	58 (65)	54 (61)	62 (70)	45 (51)

Human sera were from patients affected by colorectal carcinoma (CRC) and healthy subjects (HS). The prevalence of JCPyV antibodies in CRC patients is statistically lower than that detected in HS (**p = 0.0047).

Sera reacting to JCPyV VP1 K mimotope achieved a prevalence of 26% (14/53) in CRC, while the prevalence observed in the control group was 61% (54/89).

The same test was then exploited to investigate the IgG class of abs against the other JCPyV VP1 mimotope, the peptide N. It resulted that JCPyV VP1 mimotope N was revealed in serum samples with a prevalence of 30% (16/53) in CRC, whereas in HS the prevalence was 70% (62/89).

Interestingly, the two prevalence of IgG antibodies against JCPyV peptide N and peptide K did not statistically differ in the analyzed samples (**Table 1**).

In this investigation, JCPyV VP1-positive sera were those samples that reacted to both polypeptides K and N. Putting together the results of positive sera for both JCPyV VP1 K and VP1 N mimotopes, a prevalence of 26% (14/53) in CRC and 51% (45/89) in HS was achieved, being the different prevalence between the two groups statistically significant (p < 0.001) (**Table 1**).

JCPyV-positive sera assessed by indirect ELISA presented a general cut-off, between 0.17-0.19 OD (spectrophotometric reading). This cut-off level discriminated JCPyV-negative (sera below OD 0.17–0.19) from JCPyV-positive sera (above OD 0.17–0.19). The JCPyV hyperimmune serum, used as positive control, reached an OD of up to 1.8, whereas BKPyV and SV40 hyperimmune serum samples, the negative controls, showed an OD < 0.01. The human peptide hNPS, used as a negative control, revealed an OD below 0.1. The 2 indirect ELISAs, with 2 distinct JCPyV VP1 K and N peptides showed similar data, thus corroborating the detection of abs against JCPyV VP1 in CRC serum samples, even if at a significant lower prevalence compared to HS (**Table 1**).

**Figure 1:**
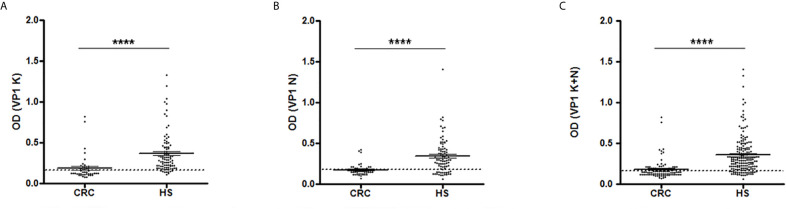
Serologic profile of human serum antibody reactivity to JCPyV viral capsid protein 1 (VP1) mimotopes. Immunologic data are from patients affected by colorectal carcinoma (CRC) and healthy subjects (HS). Results are shown as values of optical density (OD) readings at λ 405 nm for serum samples diluted 1:20 and analyzed by indirect ELISA. Each scatter plot represents the dispersion of individual OD values to a mean level, defined by the line inside the scatter with standard error of the mean (SEM) for each group patients/subjects evaluated. The mean OD for VP1 K **(A)**, VP1 N **(B)** and VP1 (K+N) **(C)** in CRC sera are statistically lower than that observed in HS (****p < 0.0001). Dotted line indicates the cut-off value.

CRC and HS serum antibody reactivity to JCPyV peptides is displayed in **Figure 1**. Scatter dot plot shows the dispersion of OD values to a mean level represented by the line inside the scatter for CRC and HS cohorts.

As shown in **Figure 1A**, the mean OD for VP1 K was 0.187 ± 0.022 in CRC sera and 0.369 ± 0.026 in HS. Similarly, the mean OD for VP1 N was 0.177 ± 0.011 in CRC samples and 0.345 ± 0.022 in controls (**Figure 1B**). The combination of both VP1 K and VP1 N data, results in a mean OD of 0.182 ± 0.012 in CRC sera and 0.357 ± 0.017 in HS subjects (**Figure 1C**), with a statistically significant difference between the two groups in all immunological data set analyzed (p < 0001; **Figure 1**).

### Prevalence of Serum Anti-JCPyV Antibody Obtained by the Haemagglutination Inhibition (HAI) Assay

We reported in previous investigations, that the indirect ELISA with JCPyV VP1 peptides specificity is confirmed by the Hemagglutination Inhibition (HAI) assay (13, 37). Indeed, HAI test detects serum specific anti-JCPyV antibodies, which are able to abolish the viral agglutination property and evaluates their titre in the sample. To this purpose, serum samples (n=89) from HS and CRC (n=53) affected patients were serially diluted from 1:16; 1:32; 1:64; to 1:128 and evaluated by HAI test.

Seroprevalence of JCPyV-positive samples, diluted 1:128, was 28% (15/53) in the CRC cohort, whereas in the HS group the prevalence was 51% (45/89). The difference of JCPyV seroprevalence between CRC and HS cohorts was statistically significant (p < 0.05) (**Table 2**). The dilution 1:128 was selected since lower dilutions include antibodies at higher level, which could give false positive results (37). Interestingly, the prevalence of JCPyV-positive samples resulted from HAI assay and indirect ELISA did not differ statistically (p > 0.05).

**Table 2:** Prevalence of serum immunoglobulin G antibodies against JCPyV assessed by haemagglutination inhibition (HAI) assay.

Human serum	N. of samples	Median age ± SD	Male (%)	N. of positive samples (%)				
				1:16	1:32	1:64	1:128
CRC	53	69 ± 12	33 (62)	43 (81)	27 (51)	25 (47)	15 (28)**
HS	89	61 ± 11	58 (65)	79 (89)	78 (88)	69 (77)	45 (51)

Human sera from patients affected by colorectal carcinoma (CRC) and healthy subjects (HS) were evaluated by HAI assay. The prevalence of JCPyV antibodies in CRC sera, diluted 1:128, is statistically lower than that detected in HS (**p = 0.0094).

## Discussion

At present, the colorectal carcinoma (CRC) is a frequent tumor, responsible of a high mortality in distinct populations. Because of its negative impact CRC is considered a significant health concern (3). The percentage of CRC incidence is currently increasing in many geographic areas, thereby representing a worldwide health problem (45). CRC arises through a multistep process characterized by several events, such as different genetic and epigenetic modifications in the human DNA, and the involvement of exogenous factors, such as lifestyle, diet, environment and viral infections (6, 7, 46). Indeed, viral agents such herpes simplex virus (HSV), human papillomavirus (HPV), Epstein-Barr virus (EBV), Cytomegalovirus (CMV) and JC polyomavirus (JCPyV) have been associated to the CRC onset. It should be recalled that these viruses are considered potential risk factors for the onset of different gastrointestinal tract tumors, such as oesophagus, stomach and colorectal cancers (47–50).

In order to investigate the putative association between JCPyV and CRC, several studies assessed JCPyV presence and/or its genes expression or seroreactivity in CRC. Overall, these reports detected JCPyV footprints in CRCs and normal mucosal tissues. Other studies reported negative data. It is possible that discordant findings could be due, at least in part, to the analysis of different markers and distinct approaches employed (7, 31).

In this investigation, sera from CRC patients and healthy subjects (HS), the control, were evaluated for their reactivity to JCPyV VP1 mimotopes using a specific indirect ELISA and HIA approach. Our immunological data indicate that specific IgG abs against JCPyV antibodies are present in both human sera from CRC patients and HS. Specifically, the overall prevalence of IgG antibodies against JCPyV VP1was 26% in CRC and 51% in HS. This result reveals that the prevalence of abs against JCPyV in sera from colorectal carcinoma patients is significantly lower than HS (p < 0.001). It should be noted that the prevalence of JCPyV-positive samples revealed by the indirect ELISA with mimotopes did not differ from that determined by HAI test. Indeed, the prevalence of JCPyV-positive sera from CRC patients and HS was 28% and 51%, respectively.

Our findings are in agreement with two earlier serological studies, where the association between JCPyV and colorectal carcinoma was investigated using ELISAs with virus-like particles (VLPs). In these prospective studies, JCPyV seroprevalence was lower in CRC cases than in controls (28, 29). However, it is worth mentioning that investigations reporting immunological results with virus-like particles or recombinant VP1 could be due to non-specific methods employed. Indeed, the high homology of VP1 amino acid sequences among JCPyV, BKPyV and SV40 polyomaviruses, may give rise to some cross-reactivity results (13, 34, 35, 51–54).

Herein, JCPyV seroreactivity was tested employing 2 distinct techniques: (i) an innovative indirect ELISA with 2 different mimotopes of the JCPyV VP1, which detected JCPyV specific IgG antibodies in CRC and HS serum samples. This innovative immunological method did not show cross-reactivity with homologue polyomaviruses, such as BKPyV and SV40; (ii) the HAI method to corroborate the JCPyV specificity and to verify the results obtained by the indirect ELISA.

Interestingly, the lower seroprevalence of IgG antibodies from CRC patients reacting to JCPyV VP1 antigens obtained in the present investigation, has also been detected in non-Hodgkin’s lymphomas (55) as well as in a cohort of patients affected by multiple sclerosis as described in our recent investigation (36).

Our immunological data suggest that CRC patients are significantly poorly responders to JCPyV VP1 antigens. It is possible that the reduced ability of CRC patients to react to JCPyV VP1 antigens could be due to their oncologic status. In addition, different exogenous factors which may contribute to the CRC onset, could also affect normal replicating cells belonging to the immune system. Numerous immune alterations were observed in CRC of different grade. Significant dysfunctions of the anti-cancer immunity of the host have been described in the CRC onset mostly depending on escape mechanisms implemented by transformed cells to establish a suitable growth environment (56–58). Tumor microenvironment, where inflammatory and immune cells acting key roles in either controlling tumor growth or promoting a chronic inflammation status, may favour the CRC progression through the induction of immune suppressive mechanisms (59, 60). For example, in advanced CRC, malignant cells recruit regulatory T cells (Tregs) which produce immunosuppressive cytokines, transforming growth factor-β (TGF-β) and interleukin-10 (IL-10) to suppress cytotoxic T cell response and maintain immune tolerance (61–63). Moreover, some subpopulations of myeloid-derived suppressor cells (MDSCs) can suppress natural killer (NK) cell cytotoxicity by blocking NK cell-mediated production of interferons (IFNs) (63). In turn, the loss of IFNs induces the infiltration of MDSCs and Tregs (64, 65), and the presence of these immunosuppressive cells limits the efficacy of cytotoxic CD8+ T lymphocytes and gives immunosuppressive features to the tumor microenvironment. In addition, Programmed Death Ligand-1 (PD-L1) which is constitutively expressed on the surface of cancer cells can bind to PD-1 inducing the suppression of immune response, suggesting the cancer cell’s attempt to escape cytotoxic immune cells (66). During CRC, different pro-inflammatory signals can enhance carcinogenesis by promoting angiogenesis and suppressing immune-mediated tumor elimination. In particular, it has been demonstrated that the activation of the transcription factor Nuclear Factor-κB (NF-κB) in the tumor site results in production of pro-inflammatory cytokines like IL-6 and IL-1, which support proliferation and survival of cancer cells (67). NF-κB also regulates the expression of tumor necrosis factor (TNF) and cyclooxygenase 2 (COX-2), which are overexpressed in an inflammatory CRC microenvironment and are involved in tumor growth promotion (68, 69). In addition to IL-6 and IL-1, other cytokines such as TGF, vascular endothelial growth factor (VEGF), C-X-C motif chemokine 3 (CXCL3), C-X-C motif chemokine 4 (CXCL4), and high mobility group box-1 (HMGB1) can be reprogrammed by tumor cells, giving rise to an immunosuppressive cytokine-dominated CRC microenvironment, which is a common mechanism for immunosurveillance escape of cancer (63). At present, it is not known if the lower prevalence of OD values for IgG against JCPyV VP1 antigens detected in CRC patients compared to HS, relies on a general or a specific impairment of CRC immune system. It is possible that JCPyV may infect colorectal cells, without being hampered by the altered immune system and transform them in tumor cells, acting as an oncogenic virus. Albeit our data suggest an inverse association between CRC and JCPyV, it is not yet clarified whether this polyomavirus is involved in the development of human CRC.

We may reason that JCPyV acts in genetically predisposed subjects when dysfunctions of the host immune system arise, because of disease-related factors, or when the immune system is not anymore fully active due to age. It is plausible that in some elderly subjects, the decline of immune system functions may favour the CRC onset and progression.

## Data Availability Statement

The raw data supporting the conclusions of this article will be made available by the authors, without undue reservation.

## Ethics Statement

The studies involving human participants were reviewed and approved by County Ethics Committee of Ferrara. The patients/participants provided their written informed consent to participate in this study.

## Author Contributions

Conceptualization: ET, IB, SP, FM and MT. Investigation and methodology: ET, IB, SP, EM. Clinical records and sample collection: GG, CF. Formal analysis and data curation: ET, IB, SP, EM, MT. Writing-original preparation: ET, MT. Supervision: MT, FM. All authors contributed to the article and approved the submitted version.

## Funding

This research was funded by Associazione Italiana per la Ricerca sul Cancro (AIRC), Milan, grant number IG 21617 (to MT); University of Ferrara, Fondo di Ateneo per la Ricerca (FAR), grants 2019-2020 to MT, FM; Fondo di Incentivazione per la Ricerca (FIR), grant 2017 to FM, and grant 2020 to EM.

## Conflict of Interest

The authors declare that the research was conducted in the absence of any commercial or financial relationships that could be construed as a potential conflict of interest.
